# A systematic review and meta-analysis of anti-epileptic medication non-adherence among people with epilepsy in Ethiopia

**DOI:** 10.1186/s13690-020-00405-2

**Published:** 2020-05-01

**Authors:** Zelalem Belayneh, Birhanie Mekuriaw

**Affiliations:** Department of Psychiatry, College of Health and Medical Science, Dilla University, Dilla, Ethiopia

**Keywords:** Anti-epileptic, Medication, Drug, Adherence, Compliance, Epilepsy

## Abstract

**Background:**

Epilepsy is the common neurological disorder in the world, affecting approximately 50 million people. Anti-epileptic medication non-adherence can be a reason for long term hospitalization, repeated emergency seizure attacks, increased health care cost and frequent absence of work due to poor seizure control. Existed studies of anti-epileptic medication non-adherence in Ethiopia have reported great discrepant and inconsistent results which calls a growing demand of systematic review and meta-analysis. Therefore, this review aimed to show the pooled prevalence of anti-epileptic medication non-adherence among people with epilepsy attending outpatient department.

**Methods:**

Literatures were searched from the PubMed/Medline, Science Direct, PsycINFO, Hinnari and Google Scholar for grey literatures. The data were extracted using a prepared data extraction Microsoft Excel format. The data were analyzed using STATA- version 14 (software). The I^2^ test was used to check the heterogeneity between primary studies with a corresponding 95% confidence interval.

**Results:**

A total of fourteen primary studies of anti-epileptic medication non-adherence were included in the review showing the pooled prevalence of anti-epileptic medication non-adherence to be 39.77 (95% CI: 32.44, 47.10). The highest prevalence [44.13 95% CI: (29.92, 58.34)] was observed among studies used both self-report and medical record review together, and studies used only self-report to screen medication none adherence had the lowest prevalence [37.95% (24.50, 51.41)]. Presence of co-morbid illness [2.27 (95%CI: 1.01, 5.12)], medication side effects [1.84(95% CI: 1.43, 2.38)], substance use or drug abuse [2.01(95% CI: 1.27, 3.20)] had statistically significant association with anti-epileptic medication non-adherence.

**Conclusion:**

In this review, we found that there is a high burden of anti-epileptic medication non-adherence among people with epilepsy in Ethiopia. This demonstrates a need for clinicians to give more attention for the monitoring and evaluation of anti-epileptic medication adherence in the health care service. We also highly recommended for the adoption of a standardized and contextualized adherence screening tools.

**Trial registration:**

**PROSPERO registration number-** [CRD42019137631].

## Background

Epilepsy is a chronic neurological disorder characterized by repeated seizure attacks resulted from paroxysmal uncontrolled discharges of neurons within the central nervous system [1–3]. Epilepsy is considered as the most common neurological disorder in the world affecting approximately 50 million people in all age groups [4–7]. The life time prevalence of epilepsy ranges from 5.8 per 1000 in developed nations to 12.7 per 1000 in rural areas of developing countries [8], but it is under recognized and untreated problem [9, 10].

Anti-epileptic drugs (AEDs) are effective in the treatment of epilepsy by controlling the occurrence of seizure attacks, and approximately 70% of people with epilepsy can become seizure-free once they start the most effective treatment regime [11]. However, evidences reported that there is huge treatment gap among people with epilepsy in low and middle income countries (LMIC) ranging from 25 to 100% [12]. The World Health Organization launched the mental health Gap Action Program (mhGAP) to address the high treatment gap for priority mental, neurological and substance use or drug abuse (MNS) disorders in LMIC and epilepsy is one of the highly prioritized disorder [13–15].

Although there are many interventions attempted to improve the anti-epileptic medication adherence of people with epilepsy, most interventions seem to fail to meet their goals and the problem of anti-epileptic medication non-adherence still exists [16, 17].

Thus, AED non-adherence is one of the common reason for the poor treatment gap of epilepsy and therefore, maintaining the adherence of anti-epileptic medication is very crucial to achieve the maximum possible therapeutic health outcomes [17–19].

In Ethiopia, there are different traditional/supernatural beliefs that can have the potential to enforce people to prefer traditional help seeking behaviour [19, 20] which may push people to miss or stop their anti-epileptic medication intake [21]. The prevalence of anti-epileptic medication non-adherence was reported to be more than expected that accounts up to 68% in Southern Ethiopia [22].

Economical constraints [23], poor health care system and medical services [24], lack of medication access [25], unrecognized anti-epileptic medication side effects, substance use or drug abuse and poor seizure control status [26] are expected to contribute for the high burden of anti-epileptic medication non-adherence in Ethiopia [22, 27, 28].

People with poor anti-epileptic medication adherence are more prone to have frequent hospital admissions, repeated seizure attacks, increased health care cost, poor quality of life, poor treatment outcome and lowered level of productivity [29–33]. It is also evidenced that anti-epileptic medication non- adherence is associated with high rate of road accidents [34], injury [35] and sudden death of patients due to the uncontrolled seizure attacks [36, 37].

Poor anti-epileptic medication adherence compounds the challenges of health status improvement among poor populations, particularly in developing countries [37, 38]. This demonstrates a need for researching evidences regarding anti-epileptic medication non-adherence and its associated factors to design appropriate strategies aiming at increasing of treatment adherence as a means to achieve the desired treatment outcome of people with epilepsy [38, 39].

Although, many studies have been existed regarding anti-epileptic medication non-adherence of people attending outpatient department in Ethiopia, there is a great discrepancy and inconsistency of reported results ranging from 21.8% [22] to 68% [40]. A full picture of magnitude of the problem is critical to develop effective intervention and for policy response that can support to improve treatment adherence of AED. This calls a growing demand of systematic review and meta-analysis regarding antiepileptic medication non-adherence and its determinants in Ethiopia. Therefore, this review aimed to show the pooled prevalence of anti-epileptic medication non-adherence among people with epilepsy attending outpatient department.

## Methods

### Reporting and protocol registration

This review followed the Preferred Reporting Items for Systematic Reviews and Meta-analysis guideline (PRISMA-P) protocol (**Additional file****1**). The review protocol has been registered in the International Prospective Register of Systematic Reviews (PROSPERO) with registration number of “CRD42019137631”.

### Search methods

Search of both published and unpublished reports of primary articles related to anti-epileptic medication non-adherence and its associated factors of people attending outpatient department was conducted. A systematic literature search was conducted using PubMed/Medline, Science Direct, PsycINFO, Hinnarri data bases, and Google Scholar for grey literatures. We also searched literatures using the direct web sites of local (Ethiopian) journals. The key terms used to retrieve primary articles were (medication OR drug OR anti-epileptic OR anti epileptic) AND adherence OR compliance OR non-adherence OR non-compliance AND Ethiopia). All important literatures available until June 3^rd^, 2019 having reports of prevalence or correlates of anti-epileptic medication non-adherence were included in this systematic review and meta-analysis.

### Eligibility criteria

The overall identified studies were exported to the End Note citation manager to avoid duplications and then, assessed for their eligibility to be included in this systematic review and meta-analysis using a prepared Micro-Soft Excel assessment format.

### Inclusion criteria

#### Study area

Research articles conducted in Ethiopia were included in this review.

#### Study design

Observational studies (cross-sectional, case-control and cohort studies) with original data reporting the prevalence or associated factors of anti-epileptic medication non-adherence were considered as eligible to be included in this review.

#### Language

Literatures written in the English language or had additional English version.

#### Population

Studies conducted among adults (age grater or equal to 18 years) with anti-epileptic treatment were included.

#### Publication issue

Both published and unpublished articles available until June 3^rd^, 2019 were included.

### Study selection

The two authors (ZB and BM), independently evaluated the eligibility of primary studies to be included in this review using PRISMA guideline **(**Additional file 1**)**. First, duplication of articles was avoided from the overall identified studies using End Note citation manager. Then, papers were evaluated by reading their titles and abstracts. In the title and abstract evaluation, studies reporting at least one of the following (anti-epileptic medication non-adherence, anti-epileptic medication adherence, odds ratio, risk ratio, or relative risk) were considered for further evaluation by full text reading. After reading the full texts of selected articles, studies fulfilling the eligibility criteria were included in this systematic review and meta-analysis. Disagreements between the two assessors were solved by re-evaluating the eligibility by both authors together.

### Outcome measurement

Main outcomes of this systematic review and meta-analysis are the pooled prevalence and associated factors of anti-epileptic medication non-adherence among people with epilepsy in Ethiopia. Anti-epileptic medication non-adherence was measured from the direct reports of the primary studies. Some studies reported the adherence level, not non-adherence of anti-epileptic medication. In this case, we used the level of AED non-adherence by subtracting the reported prevalence of adherence level from total samples. We also measured medication non-adherence from studies in which anti-epileptic medication non-adherence was measured as an explanatory variable of other outcome variables among people attending epilepsy outpatient treatment in Ethiopia.

For the second outcome, we searched factors associated with anti-epileptic medication non-adherence using words like determinants, predictors, barriers, associated factors, risk factors, reasons, correlates, and influencing factors. Crude odds ratio was calculated from primary studies to measure the strength of the association between independent variables (sex, substance or drug abuse, mental distress and medical follow-up duration) and the dependent variable (anti-epileptic medication non-adherence).

### Quality assessment

Both authors, independently evaluated the overall qualities of the primary articles using the Newcastle-Ottawa Scale for cross-sectional studies quality assessment tool [41]. The tool had different indicators consisting of three main parts. The first part of the tool had five components used to assess the methodological quality of each study. The second section examines the comparability of primary studies. The last part also measures the quality of the original articles with respect to their statistical analysis and interpretation. Any disagreements between two assessors were negotiated through discussion and by taking the average score of the two different assessment results. Articles with medium (fulfilling 50% of quality assessment criteria score) and high (6 out of 10 scores) quality were included in this review.

### Data extraction

The data extraction of this review was done using a prepared Microsoft Excel format**.** For the first objective, the data extraction format had components of first author’s name, publication year, regions of the country where the study was conducted, screening tool used, sample size, response rate and prevalence of anti-epileptic medication non-adherence. Each component was presented by a table with a single column. The findings of each primary study were presented in a single row with respect to the above mentioned components as columns. For the second objective, data extraction format prepared in a two by two table form was used **(**Additional file 2**).**

### Statistical procedures

The extracted data were imported from the Micro Soft Excel data extraction format to STATA Version 14.0 (software) for analysis. The standard error for the prevalence of ant-epileptic medication non-adherence was calculated using the binomial distribution formula for each original article. We checked the heterogeneity of primary studies using I^2^ test [42]. Based on the test result, a random-effects meta-analysis model was used to estimate the Der Simonian and Laird’s pooled effect of ant-epileptic medication non-adherence. In addition, subgroup analysis was performed based on screening tools used to measure anti-epileptic medication non-adherence to minimize the random variations between the point estimates of the primary studies. Potential publication bias had also been examined through visual assessment of the funnel plot and Egger’s test statistics at 5% significant level [43, 44]**.**

## Results

### Search results

**Fig. 1:**
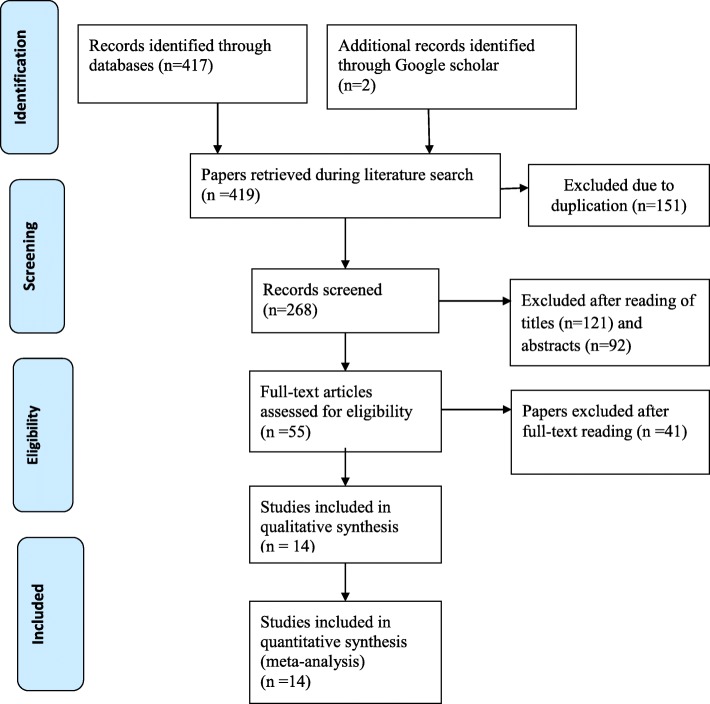
Flow chart explaining the selection of primary studies

The database and manual searches of literatures yielding a total of 419 primary articles were retrieved. About 151 articles were excluded due to duplication. The remaining 268 articles were evaluated by reading their titles and abstracts. During title and abstract evaluation, a total of 217 results were excluded and 55 articles were selected for further evaluation by reading their full texts. After full text evaluation, 41 articles were again excluded due to differences in the study population and outcome of interests. Finally, 14 primary studies were included in this systematic review and meta-analysis by fulfilling the inclusion criteria **(**Fig. 1**).**

### Original characteristics of primary studies

There were fourteen primary studies included in our systematic review and meta-analysis. These studies were conducted across different regions of Ethiopia (two primary articles from Tigray [45, 46], six at Amhara [47–52] two Oromia [53, 54], two South Nation, Nationality and People (SNNPR) [22, 55] and other two from Addis Ababa [40, 56]. The sample size of primary studies included in this review was considerably variable ranging 88 to 450 participants. Almost all primary studies have good response rate; seven studies have 100% response rate and the smallest is 78.5%. In this systematic review and meta-analysis, we performed a subgroup analysis based on the techniques or screening tools which has been used by primary studies to measure anti-epileptic medication non-adherence. Based on the Newcastle-Ottawa Scale quality assessment tool, the quality score of studies were acceptable with scores of 6 to 9 points from a total score of 10.

In this review, the pooled prevalence of anti-epileptic medication non-adherence was calculated from a total of 4018 adults attending follow-up service for epilepsy in Ethiopian using a total of 14 primary studies (Table.1).

**Table 1:** Summary of primary studies of reporting anti-epileptic medication non-adherence among adults with epilepsy in Ethiopian, 2019(*n* = 14)

First author	Publication year	Region	Assessment techniques	Total sample	Outcome	Quality assessment	Prevalence (%)
Asmamaw et al. [47]	2016	Amhara	MMAS	450	170	9	34.9%
Berhanu et al. [51]	2015	Amhara	Self report	405	130	9	32.6%
Gizachew et al. [48]	2018	Amhara	Self report	88	30	8	34.1%
Mekdes et al. [50]	2018	Amhara	MMAS	408	100	8	24.5%
Tefera et al. [52]	2001	Amhara	Self report	96	28	7	29.2%
Bereket et al. [49]	2019	Amhara	Self report	394	174	9	44.2%
Melak et al. [56]	2017	Addis Ababa	MMAS	337	119	9	30.0%
Asrat et al. [40]	2017	Addis Ababa	Self report	422	92	8	21.8%
Yirga et al. [46]	2019	Tigray	Self report and medical record review	292	191	7	65.4%
Yirga et al. [45]	2018	Tigray	Self report and medical record review	270	139	9	51.4%
Temesgen et al. [22]	2016	SNNPR	MMAS	194	132	7	68.0%
Maregu et al. [55]	2017	SNNPR	MMAS	265	101	6	38.1%
Hiwot et al. [53]	2014	Oromia	Self report and medical record review	265	98	8	36.9%
Gosaye et al. [54]	2015	Oromia	MMAS	132	61	7	46.2%

### Anti-epileptic medication non-adherence

**Fig. 2:**
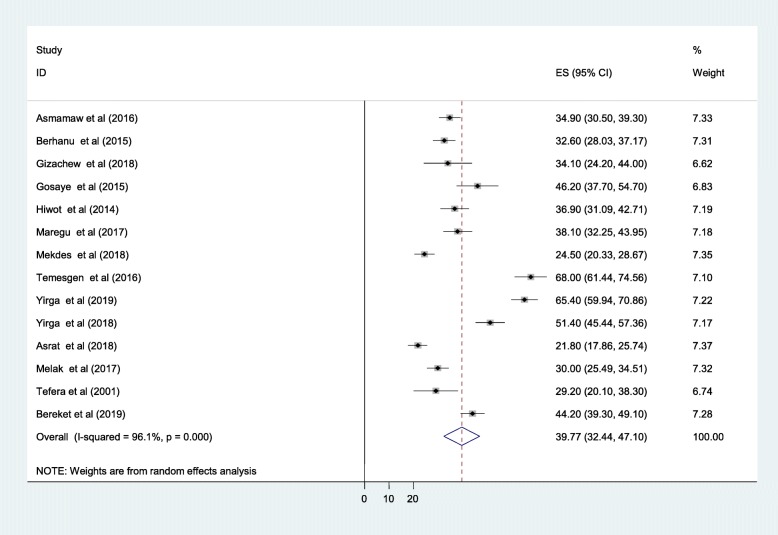
Forest plot for the pooled prevalence of anti-epileptic medication non-adherence

The magnitude of anti-epileptic medication non-adherence was varied considerably across reports of primary studies in Ethiopia. The highest prevalence of anti-epileptic mediation non-adherence was 68.0% as reported by a study conducted at Yirgalem hospital [20] and the smallest prevalence of anti-epileptic medication non-adherence (21.8%) was reported from a study conducted in Addis Ababa. The overall pooled prevalence of anti-epileptic medication non-adherence was found to be 39.77% (95% CI: 32.44, 47.10) among adults attending anti-epileptic treatment in Ethiopia (Fig. 2)**.**

### Sub-group analysis based on the screening techniques of adherence

**Fig. 3:**
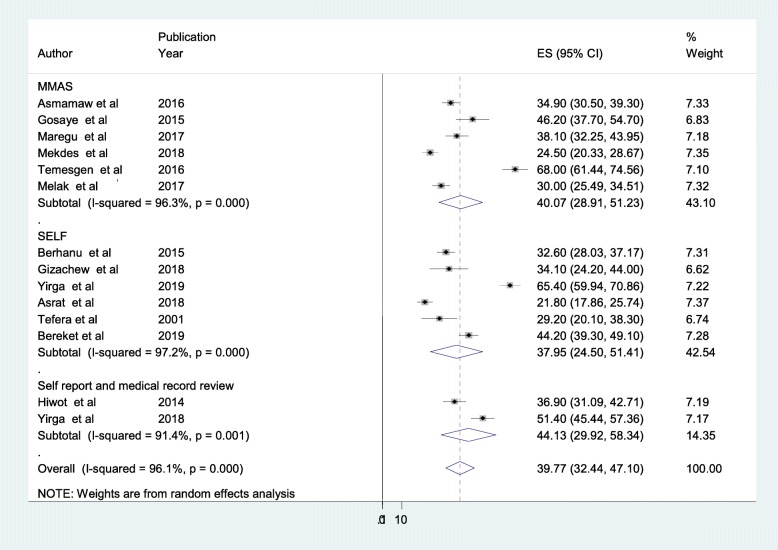
Forest plot depicting the sub-group analysis of anti-epileptic medication non-adherence

Regarding the techniques used by primary studies to measure anti-epileptic medication non-adherence, Morisky Medication Adherence Scale (MMAS) was used by six (42.85%) studies. Other six studies (42.85%) used self reporting questionnaire and the remaining two studies (14.30%) used both-self report and medical record review together. The result of this sub-group analysis indicated that there was a significant variability of between reports of primary studies regarding the magnitudes of anti-epileptic medication non-adherence depending on the difference of screening tools/techniques used to measure anti-epileptic medication adherence. Studies used both self-report questionnaire and medical record review together showed the highest prevalence (44.13%) of anti-epileptic medication non-adherence, and the lowest prevalence (37.95%) was observed among studies used only self-report questions to measure anti-epileptic medication non-adherence (Fig. 3).

### Crude analyses of determinants of anti-epileptic medication non-adherence

From the total of 14 articles included in this systematic review, seven studies reported only the magnitude of anti-epileptic medication non-adherence and other seven articles (50%) [22, 46–48, 53, 55, 56] reported both the magnitude and associated factors of anti-epileptic medication non-adherence. Although there were numerous factors being reported as predictors of anti-epileptic medication non-adherence, variables reported as associated factors of anti-epileptic medication non-adherence among at least three primary studies were considered for this meta-analysis. Accordingly, the presence of co-morbid illness, medication side effects, substance use or drug abuse and longer medical follow-up duration were included in this meta-analysis to measure their crude association with anti-epileptic medication non-adherence (Table.2).

**Table 2:** Summary of primary studies reporting correlates of anti-epileptic medication non-adherence among adults with epilepsy in Ethiopian, 2019(*n* = 7)

Authors’ name and publication year of primary studies	Determinant factors of anti-epileptic medication non-adherence											
	Sex	Educational status	Marital status	Comorbid illness^**a**^	Substance use or drug abuse^a^	Health information	Medical follow-up	Seizure control	Drug availability	Residency	Perceived stigma	AED side effect^**a**^
Asmamaw et al., 2016 [47]				√	√	√	√		√		√	√
Gizachew et al., 2018 [48]	√			√			√					√
Melak et al., 2017 [56]		√	√				√			√		√
Yirga et al., 2019 [46]				√	√			√				
Temesgen et al., 2016 [22]	√			√			√	√		√		
Maregu et al., 2017 [55]				√	√	√	√		√		√	√
Hiwot et al. 2014 [53]		√	√									√

^**a**^**Factors included in this meta-analysis**

**Fig. 4:**
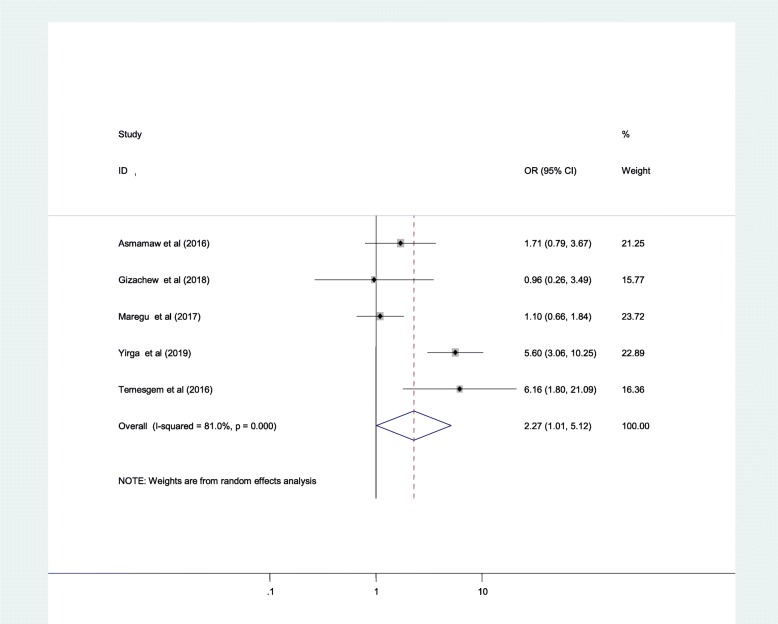
Forest plot presenting pooled random-effect size (OR) of comorbid illness

The result of the Meta analysis showed that co-morbid illness, medication side effects, substance use or drug abuse were factors significantly associated with anti-epileptic medication non-adherence while medical follow-up duration did not show significant crude association. Accordingly, individuals with comorbid illness were 2.27 times more likely to be anti-epileptic medication non-adherent as compared to their counterparts [2.27(95%CI: 1.01, 5.12)] (Fig. 4**)**.

**Fig. 5:**
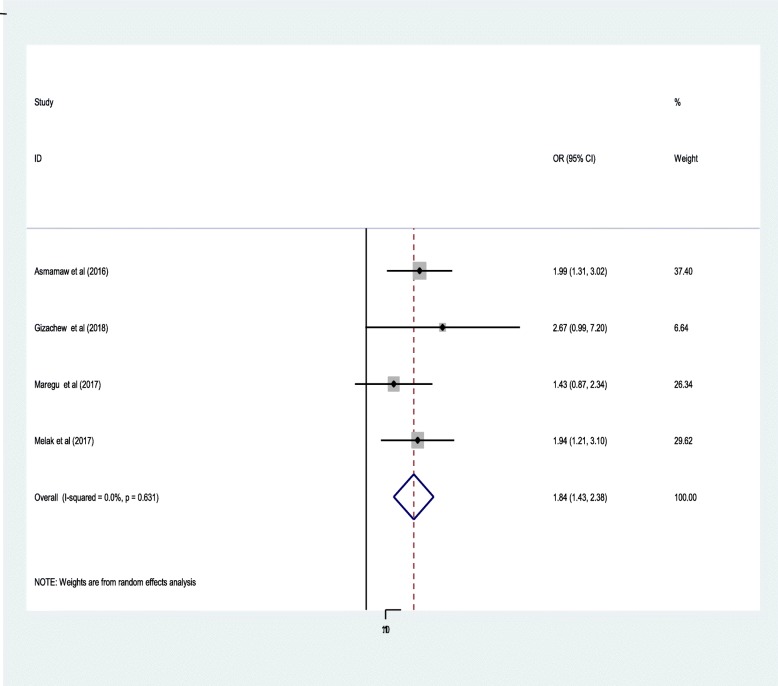
Forest plot presenting pooled random-effect size (OR) of medication side effect

The odds of having anti-epileptic medication non-adherence among adults with a complaint of anti-epileptic medication side effects were 1.64 times increased as compared to epileptic patient without complains of medication side effects [1.84(95% CI: 1.43,2.38)]**(**Fig. 5**).**

**Fig. 6:**
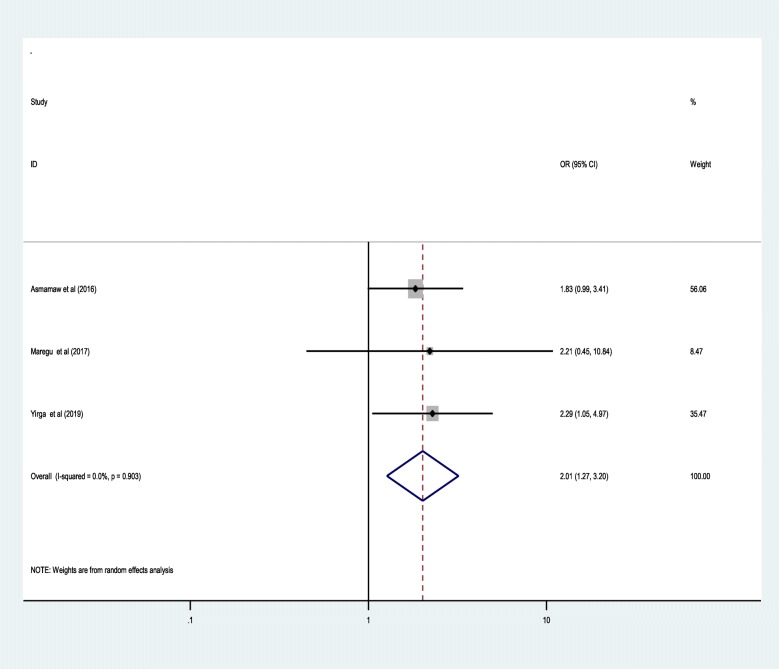
Forest plot presenting pooled random-effect size (OR) of substance or drug use

Similarly, adult epileptic patients who had substance use or drug abuse behaviour were 2.01 times more likely to be anti-epileptic medication non-adherent as compared to adults who had not current substance use or drug abuse behaviour [2.01 (95% CI,1.27, 3.20)]**(**Fig. 6**).**

**Fig. 7:**
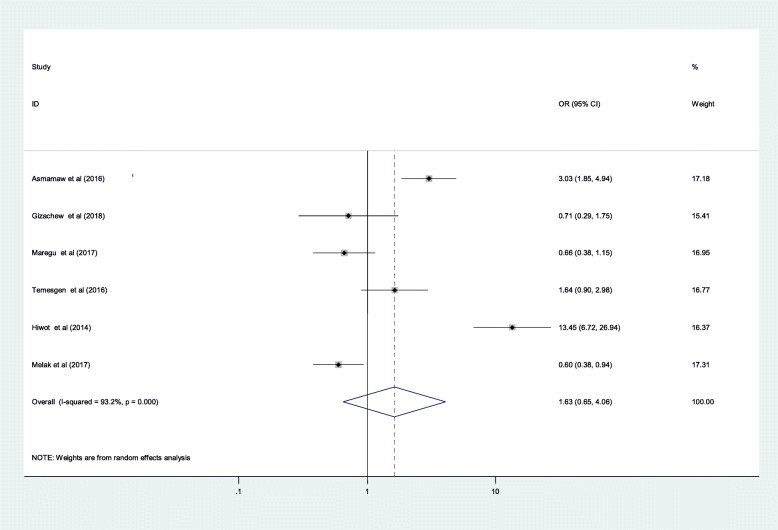
Forest plot presenting pooled random-effect size (OR) of medical follow-up duration

Finally, the Meta analysis result could not show a significant crude association between the medical follow up duration and ant-epileptic medication non-adherence [1.63 (95%CI: 0.65, 4.06)] (Fig. 7).

## Discussion

The findings of the current systematic review showed that nearly two among five (39.77%) epileptic adults are non-adherent towards their anti-epileptic medications in Ethiopia. As per the authors’ knowledge, this is the first systematic review and meta-analysis in Ethiopia to estimate the pooled prevalence and determinants of anti-epileptic medication non adherence. The result of this systematic review has a supreme importance to improve the quality of care for people suffering from epilepsy by showing the summarized figure of AED non-adherence level and suggesting possible strategies to improve the treatment adherence level of people taking anti-epileptic drugs. Moreover, the review can have clinical importance and potential policy response for health care systems of rural settings. Therefore, maintaining the adherence of the anti-epileptic medications is crucial, and also a challenging duty that needs collaborative efforts from multi-sectored dimensions [31–33].

The finding of this review showed the magnitude of anti-epileptic medication non-adherence as it is in line with another similar study conducted on medication adherence of people with epilepsy (40%) [15]. However, the finding of the current review reported a higher magnitude of anti-epileptic medication non-adherence as compared to other reviews [48, 57]. The possible explanation for the higher burden of anti-epileptic medication non-adherence in this review might be due to the more supernatural perception of epilepsy attributed by community members of Ethiopia than developed nations. Moreover, this might be due to the better availabilities of strong health care system in western nations as compared to Ethiopia.

The pooled prevalence of anti-epileptic medication non-adherence in this systematic review was inconsistence with the WHO mhGAP strategies proposed to addresses treatment gaps of MNS [14]. Thus, implementation of cell phone-based health message as reminders, counseling service and interventions targeting to improve patients’ knowledge regarding their illness and importance of medication adherence is recommended among people with epilepsy in Ethiopia [13, 21, 27, 49].

The sub-group analysis of this systematic review showed that there was a significant difference between reports of anti-epileptic medication non-adherence among primary studies based on differences regarding the screening techniques used to measure anti-epileptic medication non-adherence. Accordingly, the highest prevalence (44.13%) [95% CI: (29.92, 58.34)] of anti-epileptic medication non-adherence was observed among studies used both-self reports and medical record review together as measurement of medication adherence whereas studies used only self report showed a lower prevalence (37.95%) of anti-epileptic medication non-adherence. This discrepancy is possibly explained by the fact that people always tend to undermine or deny their treatment non-compliance, particularly medication misses while using self report as a measuring instrument for medication non-adherence [36, 58]. On the other hand, integration of medical record review with self-report questions can have a better ability to elicit the true figures of medication adherence [57]. This initiates a need to have validated, contextualized and standardized screening tools with a common definition of adherence [58, 59].

The second objective of the present study was to examine correlates of ant-epileptic medication non-adherence among adult epileptic patients in Ethiopia. The presence of co-morbid illness, medication side effects and current substance use or drug abuse behaviour were found to have statistically significant correlation with anti-epileptic medication non-adherence. Accordingly, epileptic patients with co-morbid illness were 2.27 times more likely to be anti-epileptic medication non-adherent as compared to their counterparts. This could be explained due to the fact that patients with co-morbid illness are more likely to have other medications together with their anti-epileptic medications. This may make them to be more reluctant to take all medications properly due to pills burden [7]. Moreover, the possibility of drug-drug interaction and complicated medications adverse effect may also contribute for the increased AED non-adherence of patients with co-morbid illness [54].

It is evidenced that medication side effects are significant predictors for anti-epileptic medication non-adherence. The findings of this meta-analysis also confirmed this conclusion by showing that the odds being non-adherent towards anti-epileptic medication among adults with a complaint of anti-epileptic medication side effects were 1.84 times increased as compared to epileptic patient without anti-epileptic medication side effects. This is possibly explained by the fact that anti-epileptic medications side effects can have a potential to affect both the physical and psychological states of people with epilepsy. People may also lack trust towards medications while there is serious side effect and they may stop their medication intake [21, 35, 51].

Similarly, adults epileptic patients in Ethiopia who had current substance use or drug abuse behaviour were 2.01 times more likely to be anti-epileptic medication non-adherent as compared to adults who had not current substance use or drug abuse habit. This might be due to the fact that psycho-active substances have a direct brain effect that enables individuals to be forgetful or negligence towards their anti-epileptic medication as they are often preoccupied by searching of mechanisms to satisfy their urgent needs of substance use [46].

There are contradicting evidences regarding the influence of medical follow-up duration on the medication adherence level of people with epilepsy. Some studies reported that longer medical follow-up duration of epileptic patients had a negative effect on the medication adherence level [46]. Such studies justified this correlation by the fact that people with epilepsy often show more concern and commitment towards their treatment as soon as they start first and diminish later. They also argued that anti-epileptic medication is not freely accessible in different countries and people might not afford to purchase their medication for longer duration. In the contrary, other studies justified that epileptic patients become more adherence as their medical follow-up duration becomes longer and longer due to the better seizure control status and possibility to gain more knowledge towards their illness and the importance of maintaining adherence [21, 60]. However, the finding of this review showed that there was no statistically significant correlation between medical follow-up duration and anti-epileptic medication non-adherence among adults attending outpatient department in Ethiopia [1.63(95%CI, 0.65, 4.06)]. This demonstrates a need for searching further evidences.

Generally, findings from this systematic review and meta-analysis showed that anti-epileptic medication non-adhere level of people attending outpatient department of epilepsy clinics in Ethiopia needs a collaborative effort, and professions are recommended to integrate the psychosocial support system together with the pharmacological intervention [31, 32]. This can be added as one means of addressing treatment gaps and improving quality of care for with epilepsy. Designing appropriate and contextualized screening tools of anti-epileptic medication non-adherence is also recommended.

### Strength and limitations of the study

In this systematic review, there was an extensive search of data bases and grey literatures. The quality of studies included in this systematic review was also assessed using standardized measurement, and all articles meet the specified criteria. However, the study has limitations due to the selection of only articles written or translated to English.

## Conclusions

In this review, we found that there is a high burden of anti-epileptic medication non-adherence among adults attending outpatient department in Ethiopia. There were different and non-uniform adherence measurement techniques showing a significant variation in the reported magnitudes of anti-epileptic medication non-adherence. Presence of co-morbid illness, medication side effects and substance use or drug abuse behaviour had crude association with anti-epileptic medication non-adherence. This demonstrates a need for clinicians to give more attention in the monitoring and evaluation of adherence for anti-epileptics in the health care services. We also highly recommended for the adoption of validated, contextualized and standardized medication adherence screening tools. Furthermore, prevention, early screening and intervention of co-morbid illness, medication side effects and substance abuse behaviour are vital to promote anti-epileptic medication adherence in Ethiopia.

## Supplementary information

**Additional file 1: Supplementary file 1:** PRISMA-P (Preferred Reporting Items for Systematic review and Meta-Analysis Protocols) 2015 checklist: recommended items to address in a systematic review protocol*

**Additional file 2.**

## Data Availability

All the data included in the manuscript can be accessed from the corresponding author Zelalem Belayneh upon request through the email address of“zelalembe45@gmail.com”.

## Abbreviations

AED: Anti epileptic drug
AOR: Adjusted odd ratio
CI: Confidence interval
ES: Estimates of standard error
LMIC: Low and middle income countries
mhGAP: Mental health gap action program
MMAS: Morisky medication adherence scale
MNS: Mental neurological and substance use
PRISMA: Preferred reporting items for systematic reviews and meta-analyses
SNNPR-: Southern Nation nationalities and peoples of region
WHO: World health organization
